# Valuing diagnostic AI: a structured reimbursement model for learning healthcare systems

**DOI:** 10.3389/fdgth.2025.1642750

**Published:** 2025-10-21

**Authors:** Jan Kirchhoff, Christian Schieder, Fabian Berns, Johannes Schobel

**Affiliations:** ^1^ Institut DigiHealth, Hochschule Neu-Ulm, Neu-Ulm, Germany; ^2^ medicalvalues GmbH, Karlsruhe, Germany; ^3^ Weiden Business School, OTH Amberg-Weiden, Weiden, Germany

**Keywords:** diagnostic decision support systems (DDSS), AI reimbursement framework, learning healthcare system, regulatory science, ethical AI integration, value-based healthcare, medical AI governance

## Abstract

AI-based diagnostic decision support systems (DDSS) play a growing role in modern healthcare and hold considerable promise in contributing to learning healthcare systems, settings in which clinical practice and data-driven insights are closely integrated. DDSSs are increasingly used in radiology, cardiology, laboratory diagnostics and pathology, where they assist clinicians in interpreting complex data, standardized decision making, and improving outcomes. However, despite their clinical relevance, such systems remain difficult to evaluate and integrate within current reimbursement structures. Traditional key performance indicators (KPIs), such as case costs, turnaround times, or documentation completeness, are insufficient to capture the nuanced contributions of AI systems to clinical value and learning cycles. As a result, DDSS often operate outside established reimbursement logics, limiting their broader adoption and sustainability. This article addresses the economic and regulatory disconnect between the measurable value of AI-assisted diagnostics and their lack of inclusion in existing reimbursement frameworks. It introduces a structured, point-based reimbursement model specifically designed to support the integration of DDSS into real-world payment systems, using the German and American coding systems as reference models. By linking reimbursement levels with diagnostic complexity and degree of contribution from AI, the proposed framework promotes fair compensation, encourages meaningful use, and supports responsible clinical deployment. We document a multi-criteria point calibration which is anchored to existing codes. In addition, the model fosters an auditable feedback-driven structure that could support adaptive payment in learning healthcare systems. In this way, the framework is not merely a pricing tool; it also serves as a governance mechanism that aligns economic incentives with ethical, clinical, and operational priorities in AI adoption. It contributes to the realization of a learning healthcare system by enabling continuous refinement, transparent valuation, and sustainable implementation of AI-driven diagnostics.

## 1 Introduction

AI-based Diagnostic Decision Support Systems (DDSS) are increasingly at the forefront of innovation in clinical care. Using recent advances in machine learning, medical data integration, and cloud computing, DDSS aims to enhance clinical reasoning, reduce diagnostic uncertainty, and improve efficiency in specialties such as radiology, cardiology, pathology, and laboratory diagnostics. These systems analyze structured and unstructured data, including laboratory values, imaging results, and patient history, to provide differential diagnoses, risk scores, or triage suggestions that support clinician decision making. More than 1,000 AI-enabled medical tools have now been approved globally, with the United States Food and Drug Administration (FDA) alone listing hundreds of AI-supported systems for diagnostic use (1). However, despite technical readiness and growing clinical relevance, financial integration into routine care remains severely limited.

The core issue lies in the lack of reimbursement pathways for DDSS. Most AI-based diagnostic tools operate outside the established medical reimbursement frameworks, including Germany’s “Gebührenordnung für Ärzte” (GOÄ) and “Einheitlicher Bewertungsmaßstab” (EBM), or the U.S.-based Current Procedural Terminology (CPT) codes. Without specific reimbursement codes or payment models, DDSS technologies cannot be monetized in standard clinical settings, creating a structural mismatch: they deliver measurable clinical value but lack a path to financial sustainability. In Germany, this issue is particularly acute in fields like laboratory medicine and pathology, where diagnostic services are reimbursed via bundled or capped flat rates, often omitting compensation for cognitive, interpretive, or consultative services that AI may support or replicate. GOÄ and EBM currently do not provide financial mapping for AI-generated results, algorithmic pattern recognition, or longitudinal data synthesis, rendering traditional license or token-based pricing models inapplicable.

This misalignment not only slows the adoption of DDSS but also creates disincentives for hospitals and outpatient providers to invest in AI infrastructure. The clinical benefits of DDSS—earlier disease detection, improved diagnostic accuracy, and workflow standardization—are difficult to express in direct financial terms. Their effects are often indirect, delayed, and dependent on institutional variables such as staff turnover, care protocols, or documentation quality (2). As a result, return-on-investment (ROI) calculations remain elusive, particularly in reimbursement systems that lack granularity for innovation assessment. This further exacerbates the “last mile problem” of AI in medicine: Despite growing evidence of efficacy and safety, DDSS tools fail to reach the point of care due to financial, rather than technical or regulatory constraints (3).

The reimbursement gap is not limited to Germany. In the United States, for example, the Centers for Medicare & Medicaid Services (CMS) has introduced a handful of AI-related CPT codes, such as 92,229 for autonomous retinal imaging, or temporary additional payments for AI-assisted stroke detection (4). However, these approaches remain limited in scope and do not provide a scalable template. As Abrámoff et al. (5) and others argue, sustainable reimbursement must be transparent, performance aligned, and shaped through multistakeholder consensus. Emerging policy initiatives such as the US Health Tech Investment Act seek to create provisional payment frameworks for AI services, while France’s “forfait innovation” and Germany’s selective contracts offer temporary funding for digital tools, although none of these efforts address the core need for structured and operational billing logic.

In response to this challenge, the present study proposes a novel pricing framework for DDSS that is explicitly designed to integrate into the existing logic of medical billing systems. Rather than relying on external license agreements or opaque value-based pricing, the framework introduces standardized *AI contribution levels*, each tied to a defined scope of automation, clinical responsibility, and human oversight. These levels are assigned to a point-based reimbursement logic modeled after the GOÄ and EBM structures. In contrast to flat license fees or usage-based pricing models, this approach enables precise and auditable billing, facilitates payer trust, and ensures proportional remuneration aligned with diagnostic impact. The framework includes a reference billing table that covers representative domains, such as laboratory diagnostics, ECG interpretation, pathology, and radiology, and supports multiplier-based international adaptation.

This structured reimbursement logic is designed to be both scalable and interoperable. By providing a clinically anchored and policy-compatible pricing method, it addresses the shortcomings of previous models, including abstract token-based concepts or outcome-contingent schemes that often lack practical implementability. In doing so, it contributes to a more transparent and equitable integration of AI into routine diagnostics, supporting not only high-autonomy applications (e.g., automated screening) but also lower-tier assistive tools (e.g., diagnostic triage or structured reporting support).

To evaluate the feasibility and impact of the proposed pricing model, we performed an empirical analysis using representative clinical scenarios. We examined how incorporating the AI decision support fee would affect stakeholders under current reimbursement rules: the additional cost to payers, the revenue to AI developers or providers, and potential savings from improved diagnostic outcomes. AI-based diagnostic tools can contribute to a continuously learning healthcare system, where patient data and algorithmic insights are continuously fed back into care practice. Section 2 of this paper details the materials and methods of our study, including the data sources, assumptions, and analytical methodology. Section 3 presents the results of the evaluation, demonstrating the economic and operational outcomes of applying the novel pricing approach in practice. Section 4 provides a discussion of the findings, situating our proposed model in the broader context of AI reimbursement policy and technology adoption.

## 2 Materials and methods

This section outlines the research design and methodological approach used to develop and evaluate the proposed pricing framework. We describe the materials and data sources that informed our model, as well as the steps taken to evaluate how the new pricing approach performs under current billing conditions. The aim is to provide a transparent account of how the pricing scheme was constructed and tested, allowing others to understand the scope and limitations of our evaluation.

### 2.1 Methodology

In this subsection, we present the methodology employed to formulate the novel pricing model for the diagnostic decision support system and to examine its impact. We first explain the conceptual design of the pricing approach, including the assumptions made about cost components, clinical workflow integration, and payer reimbursement rules. Next, we detail the analytical procedures used to evaluate the performance of the model: this includes any simulations, cost-benefit analyses, or case studies conducted to compare the outcomes of the current billing scenario vs. the scenario with the AI pricing in place. We also specify the metrics by which the approach is assessed (for example, changes in provider revenue, payer expenditure, or diagnostic accuracy/cost trade-offs) and discuss how these metrics align with our objectives of sustainability and incentivizing adoption. By clearly delineating our methodology, we ensure that the evaluation of the pricing approach is rigorous and reproducible.

### 2.2 Evaluation of re-imbursement

This section provides a comprehensive analysis of how physician diagnostic services are currently reimbursed in Germany under the GOÄ (“Gebührenordnung für Ärzte,” or Medical Fee Schedule for Private Patients) and EBM (“Einheitlicher Bewertungsmaßstab,” Uniform Value Scale for Statutory Patients). These frameworks define the economic valuation of diagnostic activities such as radiographic image interpretation, ECG and ultrasound reporting, laboratory result evaluation, and histopathological analysis. Understanding the specific reimbursement codes and constraints within GOÄ and EBM offers a realistic baseline for determining the economic value of AI-based diagnostic decision support systems (DDSS). This section translates complex reimbursement structures into a standardized English framework and analyzes where AI can act as a supportive, assistive, or substitutive element in clinical diagnostics. Implications for pricing strategies for AI solutions in medicine are derived accordingly.

#### 2.2.1 Radiological diagnostics

Radiology is a medical discipline that relies heavily on imaging technologies, including X-rays, computed tomography (CT), and magnetic resonance imaging (MRI), to diagnose and monitor a variety of conditions. In the German healthcare system, the remuneration of radiological services is governed by two parallel reimbursement schemes: the “Gebührenordnung für Ärzte” (GOÄ) for privately insured patients and the “Einheitlicher Bewertungsmaßstab” (EBM) for those covered by statutory health insurance (GKV). These frameworks codify not only the technical procedures but also the interpretations of the associated physician, providing a quantifiable proxy for the value of radiological diagnostic work.

Under the GOÄ, radiological procedures are reimbursed based on a points system, with one point equivalent to approximately €0.058. This allows physicians to bill for individual services using a multiplier (commonly between 1.0 and 2.3 times) depending on effort and complexity. For instance, a single-plane chest X-ray (GOÄ code 5135) is valued at 280 points, resulting in a base reimbursement of roughly €16.32. More advanced imaging techniques attract higher fees: a cranial CT (GOÄ 5370) garners 2,000 points (€116.57), while a head-and-neck MRI (GOÄ 5700) may reach 4,400 points (€256.46) at the baseline rate. The actual remuneration can increase significantly with applicable multipliers or if additional services such as contrast media (GOÄ 5377) are included.

In contrast, the EBM adopts a point-based system with a fixed point value (approximately €0.12 as of 2025). Radiological services are categorized in Chapter 34 of the EBM and assigned a specific “Gebührenordnungsposition” (GOP). A chest X-ray (GOP 34240) earns 82 points (€10.16), and a cranial CT (GOP 34310) receives 534 points (€66.20). MRIs and other high-complexity procedures are compensated at higher rates but generally remain below GOÄ levels. In particular, each EBM position includes not only the technical execution of the imaging but also the mandatory interpretation and report of the physician.

A unique feature of the EBM is the introduction of teleconsultation codes, such as GOP 34810 (tele-radiological interpretation of X-rays, €13.20) and GOP 34820/34821 for standard and complex CT consultations, respectively. These positions specifically reward radiological expertise applied in cross-institutional settings without requiring direct image acquisition.

From an AI pricing perspective, radiology represents a key domain for automation. Deep learning models have demonstrated proficiency in identifying radiological anomalies, such as pulmonary nodules or bone fractures, with precision comparable to that of human radiologists. Given the standardized nature of imaging studies and well-documented reimbursement rates, the economic value of AI-supported radiological diagnostics can be quantified more precisely than in many other specialties. For example, an AI tool that reliably interprets chest radiographs might be equated to a service valued at €10–€18 per study (GKV) or €16–€26 per study (GOÄ).

However, a full replacement of radiologists remains unlikely in the near term. Instead, AI systems are more plausibly deployed as second readers, helping with triage or quality assurance. The legal and clinical obligation for the final interpretation and generation of reports will continue to reside with licensed medical professionals, underscoring the supportive rather than substitutive role of AI in radiology. As such, pricing models for AI-based radiological interpretation should reflect not only the economic value of the delegated task but also the shared responsibility framework under which these technologies operate.

#### 2.2.2 Cardiological diagnostics

The field of cardiology involves a variety of diagnostic services, including electrocardiograms (ECG) and cardiac ultrasound (echocardiography). These services are crucial for detecting arrhythmias, ischemic changes, or structural heart disease. In the German private health insurance system, known as the *Gebührenordnung für Ärzte* (GOÄ), each of these diagnostic services is assigned a specific billing code with a corresponding number of points that translate into a monetary value. Examples include GOÄ 650 for a basic rhythm check ECG, GOÄ 651 for a standard 12-lead ECG, and GOÄ 652 for a stress ECG, with values ranging from approximately €9 to €26.

Similarly, echocardiographic diagnostics are covered under GOÄ codes 422 to 424, with the complexity and comprehensiveness of the examination increasing the fee. For instance, GOÄ 422 (one-dimensional echo) is valued at approximately €12, whereas GOÄ 424 (Doppler echocardiography including two-dimensional imaging) may be billed for up to €41.

Under the statutory health insurance billing scheme, the *Einheitlicher Bewertungsmaßstab* (EBM), these services are handled differently. Routine diagnostics such as a resting ECG may not be reimbursed separately in primary care but are included in the base consultation fee. More complex tests like stress ECGs (GOP 03321), Holter ECG recordings (GOP 03322), or computer-assisted evaluation of long-term ECGs (GOP 03241) are reimbursed individually. Their monetary value varies, typically ranging between €11 and €38, depending on complexity and specialization.

For AI systems, these services represent clear opportunities. Algorithmic analysis of ECG waveforms, rhythm recognition, or even automated interpretation of echocardiographic parameters (e.g., ejection fraction, valve function) are tasks increasingly within the scope of modern AI systems. In fact, the EBM already recognizes the value of algorithmic support, with code GOP 03241 specifically reimbursing computer-aided ECG evaluation.

From a pricing perspective, AI systems that can reliably automate or augment cardiological diagnostics may be assessed in analogy to existing GOÄ and EBM codes. A basic ECG interpretation may be valued at €10–€15 under the GOÄ, whereas more complex, long-term analyses involving trend interpretation or pattern recognition might justify higher remuneration in line with GOÄ 652 or EBM 03322. However, any monetization model must consider that not all such services are separately reimbursable under the EBM. Consequently, economic value can be derived from saved physician time or improved diagnostic performance, rather than direct billing substitution.

#### 2.2.3 Laboratory diagnostics and pathology

Diagnostic services in laboratory medicine and pathology play a central role in clinical decision making. They form the basis for disease detection, therapy planning, and outcome monitoring across virtually all specialties. Despite overlapping regulatory frameworks in the German healthcare system—primarily the GOÄ for private care and the EBM for statutory insurance—the economic valuation, billing logic, and documentation standards differ significantly. This section outlines the reimbursement logic and AI applicability for each discipline in a structured manner.

Laboratory diagnostics encompass high-throughput analytical procedures including clinical chemistry, hematology, immunology, and microbiology. Under GOÄ, individual assays (e.g., glucose, CRP, complete blood count) are billed using section M codes with fixed point values (e.g., GOÄ 3560 for glucose, €2.33). The multiplier mechanism allows for adjustment based on complexity, and additional interpretative tasks may be billed separately (e.g., GOÄ 80 for narrative commentary).

In contrast, the EBM consolidates tests into flat-rate packages (e.g., Chapter 32), limits daily billable volume per specimen type, and rarely reimburses interpretative efforts. Routine lab diagnostics thus yield modest reimbursements per test (e.g., €0.25 for glucose, GOP 32057), with interpretation bundled into consultation fees. As a result, while GOÄ supports billing for interpretive or consultative services, the EBM does not incentivize advanced data analysis.

For AI-based systems, the value proposition lies in augmenting physician interpretation: synthesizing multi-parameter panels, flagging pathological constellations, or tracking trends over time. While such functionality mirrors human pattern recognition, direct reimbursement pathways remain limited, especially in the EBM setting. Nonetheless, AI-generated structured interpretations could analogously match GOÄ codes between €9–€26, depending on complexity and format.

Pathology involves tissue-based diagnostics via histology, cytology, and immunohistochemistry. In the GOÄ, services such as GOÄ 4800 (basic histology) or GOÄ 4810 (complex tumor pathology) provide the baseline for reimbursement (€12–€17 per slide), with higher total compensation resulting from additive procedures (e.g., immunostains, GOÄ 4820). Pathologists often assess multiple slides per case, leading to substantial cumulative reimbursement. EBM codes (e.g., GOP 19310 for basic histology, €10.30) offer a similar structure but at reduced rates, with interpretation assumed to be included in the analytical service.

Digital pathology opens new avenues for AI integration. Image recognition algorithms can classify malignancies, assess resection margins, or quantify biomarkers. While these tools may streamline workflows and support diagnostic accuracy, they are unlikely to replace human pathologists in the short term. Economically, AI-assisted analysis may be benchmarked against existing GOÄ or EBM slide-based codes (€10–€20), depending on diagnostic depth. Additional value comes from efficiency gains, triage automation, and quality assurance, although these are not yet reflected in formal reimbursement schemes.

### 2.3 Foundation for AI-assisted reimbursement codes

This section describes the AI Score model developed as the methodological basis for a structured reimbursement framework for AI-assisted diagnostics. The model evaluates AI Diagnostic Decision Support Systems (DDSS) along four qualitative dimensions, each scored on a 1–5 scale based on defined sub-criteria. The dimensions—(1) Data Complexity and Diversity, (2) Disease Complexity, (3) Complexity of the Clinical Question, and (4) Degree of Involvement of AI—collectively capture the clinical and technical value that an AI tool contributes to diagnostic decision-making. Below, we justify each scoring dimension in terms of real-world diagnostic support and its relevance to the reimbursement value chain. We then explain how the outcomes of this scoring inform the need for transparent, standardized reimbursement codes. This AI score model supports the proposed structured reimbursement framework by linking nuanced technical evaluation with practical payment categories.

#### 2.3.1 Data complexity and diversity

This dimension assesses the complexity of input data that the AI system must handle (e.g., imaging, laboratory results, genomic data, free-text clinical notes) as well as the diversity and quality of those data (e.g., multi-center or multi-population data vs. homogeneous data). In real-world diagnostic support, AI systems increasingly integrate multiple heterogeneous data sources, for example, combining medical images, biosensor readings, electronic health records, and genomic information in a single analysis. Managing high data complexity (such as multimodal input or high-resolution data) typically requires more sophisticated algorithms and infrastructure, reflecting greater technical value. Likewise, data diversity is crucial: An AI trained and evaluated on broad and diverse datasets is more generalizable and reliable across patient populations. Studies have noted that many AI models perform poorly in practice because training data did not capture the variability present in real clinical settings. For example, differences in imaging protocols or patient demographics between hospitals can significantly degrade the accuracy of an algorithm if not taken into account (6).

Ensuring a wide representation of data (different centers, populations, and edge cases) mitigates bias and improves robustness, directly impacting the quality of diagnostic support. In the context of reimbursement, increased data complexity and diversity often translate to greater development and operational costs (for data integration, cleaning, and storage), as well as higher clinical value (through improved accuracy and equity of the AI tool). Therefore, this dimension is a key part of the AI Score: it rewards systems that tackle complex, multifaceted data and successfully leverage diverse inputs, since such capabilities enhance diagnostic performance and ultimately patient outcomes. In the reimbursement value chain, capturing data complexity/diversity in the score helps signal when an AI service involves significant technical resources or offers broad utility—factors that payers may consider in valuing and pricing the service.

#### 2.3.2 Disease complexity

The second dimension, Disease Complexity, evaluates the intrinsic complexity of the medical condition or diagnostic problem addressed by the AI. Not all diseases are equal from a diagnostic point of view: some have straightforward and well-characterized presentations, while others are rare, multifactorial, or have nonspecific symptoms. For example, certain diseases present with ambiguous or variable clinical characteristics that require extensive examination and specialist interpretation, whereas other conditions have distinct and easily recognizable patterns that make diagnosis relatively simple. The complexity of the disease encompasses factors such as the rarity of the condition, the number of potential differential diagnoses, the variability in patient presentations, and the extent of work (and expertise) traditionally required to arrive at a diagnosis. From a real-world perspective, AI support in high-complexity diseases (e.g., diagnosing an atypical autoimmune disorder or a multiorgan syndrome) can be especially valuable—these are scenarios where human clinicians might ordinarily consult multiple specialists or conduct numerous tests. An AI DDSS that can help in such cases (by synthesizing many data points or highlighting patterns that span disciplines) contributes significant clinical value by potentially shortening the time to diagnosis or reducing misdiagnosis rates. In fact, the level of clinical experience needed to correctly label and diagnose a case correlates with the complexity of the disease.

Relevance to reimbursement: Incorporating disease complexity into the AI Score ensures that the reimbursement framework recognizes the clinical difficulty and value of what the AI is addressing. Payers are more likely to reward technologies that solve challenging problems (especially if those problems currently incur high costs or risk in the healthcare system). A higher score in Disease Complexity signals that an AI service is applied to a diagnostically challenging condition, justifying a higher reimbursement due to the specialist-level support it provides. Moreover, by distinguishing simple vs. complex disease contexts, the model aligns with the value-based care principle that interventions should be reimbursed in proportion to the complexity of care needs they meet.

#### 2.3.3 Complexity of the clinical question

Beyond the disease itself, this dimension considers the specific clinical question or task the AI is intended to answer. The scope and complexity of the question can vary widely. An AI might be asked a narrow question (e.g., “Does this chest radiograph show pneumonia: yes or no?”) or a broad one (e.g., “What is the likely diagnosis for a patient with these multiple symptoms and test results?”). It might perform a simple classification (identifying the presence of a known finding) vs. a higher-order reasoning task (providing differential diagnoses or even suggesting management options). The AI Score captures this by rating how complex the question is, with higher scores for AI tools that address open-ended or multi-step diagnostic questions, as opposed to binary or well-bounded tasks. In real-world diagnostic support, the complexity of the question strongly influences the cognitive effort and algorithm sophistication required. For example, an AI that merely flags potential tumors on a mammogram addresses a relatively straightforward question of detection. In contrast, an AI that stages a cancer (determining tumor extent and progression) or stratifies a patient;s risk and suggests the next diagnostic steps is handling a far more complex query. The latter must integrate more data points and interpret them in the context of disease progression, similar to a comprehensive clinical reasoning process. In fact, the data requirements and model design will scale with question complexity: a more complex clinical question often demands more input data and advanced logic, as noted by researchers observing that the necessary breadth of training data increases with the complexity of the diagnostic question being addressed.

By explicitly scoring the complexity of the clinical question, the model reflects the technical and clinical challenge that AI faces. This has direct implications for reimbursement. When an AI system answers a complex question (for example, guiding diagnosis in an undifferentiated patient case or performing a comprehensive image analysis with clinical context), it effectively provides a service closer to an expert consult or an integrative diagnostic work-up. Payers and health systems may assign greater value to such comprehensive decision support, as it has potential to improve outcomes (e.g., faster correct diagnoses, fewer unnecessary tests) in ways a simpler tool would not. Thus, a high score in this dimension supports the case for a distinct and higher reimbursement category. It ensures that AI tools are not treated with a one-size-fits-all approach in billing: instead, those addressing complex clinical questions can be recognized with appropriate compensation, incentivizing development of AI that tackles the most challenging diagnostic dilemmas.

#### 2.3.4 Degree of involvement of AI

The fourth dimension measures how extensively the AI system is involved in the diagnostic process, i.e., the balance of work done by the AI vs. the human clinician. This ranges from minimal assistance to full autonomy. At the lower end of the scale (score 1), AI could function in an “assistive” role, simply identifying or triaging data for the physician’s attention without performing any independent analysis or decision-making. For example, an AI that highlights abnormal areas in an image or retrieves relevant patient data points is assistive. In the mid-range, AI takes on an “augmentative” role: Performs a substantive analysis and provides a clinically meaningful output, but the physician remains fully in charge of interpretation and decision making. An example would be an AI that analyzes a cardiac CT scan and quantifies plaque burden or valve function. It gives the doctor useful analytical results, but incorporates them into the final diagnosis or treatment plan. At the highest end (score 5), AI could be “autonomous,” interpreting data on its own and even making direct diagnostic or triage recommendations. A real-world example is an FDA-approved autonomous AI that reads retinal images and diagnoses diabetic retinopathy without immediate input from the clinician. In such cases, the AI essentially performs a diagnostic task traditionally performed by a specialist.

This dimension is crucial to reflect the contribution of AI to the service. Higher involvement of AI often correlates with greater technical complexity (developing an autonomous AI is more difficult and requires more validation) and changes in workflow (the clinician’s role changes from active analysis to supervisory oversight of AI). It also raises important considerations in liability and trust—for instance, fully autonomous systems require rigorous performance guarantees before clinicians and payers will trust them in practice. From a reimbursement perspective, the degree of AI involvement has direct bearing on how a service is billed and valued. Health economic frameworks and coding authorities are beginning to acknowledge this. The CPT editorial panel of the American Medical Association, for example, introduced an AI taxonomy in Appendix S of the code set precisely to define how much of a procedure is performed by AI vs. a physician. The rationale is that differentiating machine work from human work “creates a path to payment” for AI-related services. In line with this, our AI Score’s involvement dimension ensures that an AI-heavy service can be elevated for separate reimbursement if appropriate. If an AI is doing most of the analysis (such as autonomous AI diagnostics), the value chain may treat the AI’s output like that of a specialist—worthy of its own billing code or fee. In contrast, if AI is only marginally involved, it may not warrant additional reimbursement and could be bundled into existing clinical fees. By quantifying AI involvement, the model provides a transparent basis for these distinctions. This protects against scenarios where providers may overstate the role of an AI to seek higher payment, and, conversely, it rewards genuine innovation where AI substantially increases or replaces the effort of the physician in diagnosis. In summary, the degree of involvement dimension ensures that the AI Score model is sensitive to the substitution or increase in work that occurs with AI, a critical factor for the ethical integration of AI into clinical practice and to calibrate payment to the service rendered (7).

### 2.4 From AI score to standardized billing codes

Although the AI score provides a nuanced and multidimensional evaluation of an AI diagnostic system, reimbursement systems require a translation of this score into standardized billing codes. Payers and providers operate in a framework that demands trust, predictability, and transparency. In practice, this means that a service is reimbursed via a published code (for example, a CPT code or similar) with a clear definition and a fixed payment rate, rather than by recalculating a bespoke score for each use. There are several reasons for favoring fixed codes derived from score ranges over dynamic scoring in billing:

Transparency and Consistency: A published code is a publicly defined descriptor of a service which all stakeholders understand. If the AI score were used directly to calculate reimbursement on the fly, it could introduce opacity and variability (for example, small changes in the score could alter payments and the scoring process could be viewed as a “black box”). In contrast, mapping score ranges to codes yields a finite set of discrete tiers—for instance, an AI Score in a certain range corresponds to a specific billing code—which can be transparently listed with their criteria. This approach mirrors how complexity is handled in existing coding (such as different levels of evaluation and management codes based on complexity tiers). The predictability of a fixed code builds trust: clinicians know in advance how their use of a given AI will be billed, and payers know what they are paying for without having to audit the algorithm’s scoring calculation each time.

Reliability and Auditability: Published codes undergo rigorous definition and valuation processes (often involving clinical specialty societies, regulators, and payers). Once established, using a code is straightforward to document and audit. If reimbursement were tied to a continuous AI Score, every claim would require verifying the score computation, opening the door to disputes or gaming. Standard codes remove this uncertainty; for example, an autonomous AI retinal screening now has a permanent CPT code (92229) with an assigned reimbursement, after demonstrating its safety and efficacy. This code-based approach has already enabled Medicare to reimburse the national Medicare reimbursement for that AI service, showing how a fixed code can legitimize and streamline AI payments. The existence of a code signals to all payers that the service is recognized and valued, whereas a custom score would lack that official recognition.

Avoiding unwanted incentives tying payment directly to a dynamic score could perversely incentivize providers or vendors to maximize the score (e.g., by upselling the complexity of a case) to receive higher payment, similar to how per-use or volume-based payment can encourage overutilization. In contrast, a system of tiered codes promotes fair use: each code corresponds to a bracket of AI Score that has been deemed a justifiable level of complexity/value. Providers cannot easily exaggerate the complexity beyond what the documented clinical scenario supports, and payers can require evidence that the case met the code’s criteria. This fosters trust that reimbursement is justified by the real attributes of the service, not by opportunistic scoring. In fact, experts have cautioned that without careful design, paying for AI per use could lead to overuse or misuse. A structured code system grounded in the AI Score helps mitigate these risks by setting predefined categories of AI assistance that are eligible for reimbursement.

Alignment with Existing Billing Infrastructure: Healthcare billing is built around coded entries, from procedure codes to diagnosis codes, which feed into electronic claims systems. By converting the AI Score outcomes into a code (or a combination of codes), we ensure seamless integration into billing workflows. This approach is analogous to how new medical technologies are introduced: initially, temporary codes (e.g., CPT Category III) might be used to track usage, and eventually permanent codes are established once criteria and value are agreed upon. Our framework follows this paradigm, using the AI Score model to define the criteria for new AI billing codes in a consistent manner. In effect, the score is the methodological foundation, and the codes are the implementation mechanism (4).

In conclusion, the rich evaluation of the AI Score model is distilled into a structured set of reimbursement codes that correspond to ranges or combinations of dimension scores. This ensures that while we capture the full clinical and technical value of an AI DDSS (through the four dimensions), the interface to the reimbursement system remains practical and trustworthy. Published codes grounded in this model offer the necessary predictability for providers (to know when and how they will be paid for using AI in care) and for payers (to standardize payment and budget impact). By establishing this scoring methodology and its translation into codes, we lay the foundations for a standardized AI reimbursement framework that can be broadly adopted rather than a case-by-case negotiation. This approach ultimately supports transparency in how AI-driven services are valued, fosters stakeholder trust in AI in healthcare (since reimbursement will be tied to clearly defined criteria), and encourages the adoption of truly beneficial AI innovations by ensuring they are appropriately reimbursed. Thus, the AI Score model serves as the methodological foundation for our proposed structured reimbursement framework, bridging the gap between the nuanced evaluation of AI-assisted diagnostics and the practical requirements of healthcare billing systems.

### 2.5 Point calibration

We calibrated point values using a transparent rubric that reuses the four dimensions introduced in this paper (data complexity/diversity, disease complexity, clinical-question complexity, AI involvement). Each AI service received a score from 1 (low) to 5 (high) on each dimension, based on brief anchors:

• **Data complexity/diversity:** 1 = single, low-variance data source; 5 = multi-modal/high-resolution data across diverse sites/populations.
• **Disease complexity:** 1 = common, well-delimited condition; 5 = rare/multifactorial with broad differential.
• **Clinical-question complexity:** 1 = narrow/binary detection; 5 = open-ended reasoning (differential diagnosis, next-step guidance).
• **AI involvement:** 1 = assistive pre-interpretation/triage; 5 = near-autonomous diagnostic report with minimal real-time human input.

We then computed a simple average of the four 1–5 ratings (no weights) and assigned the nearest canonical point tier from a small, fixed set to avoid over-fitting: We assign canonical point tiers based on the rounded average of the four dimension scores as follows: an average of 1–2 corresponds to **50 points** (assistive); an average of 3 corresponds to **100 points** (augmentative); and an average of 4–5 corresponds to **150 points** (high-complexity augmentative or autonomous).

To keep tiers intuitive across specialties, we anchored 100 points to two widely referenced comparators: CPT 92229 (autonomous retinal screening) and EBM 03241 (computer-assisted Holter analysis). Services clearly below these anchors in combined complexity/involvement were mapped to 50 points (e.g., assistive pre-interpretation), while services clearly above were mapped to 150 points (e.g., whole-slide pathology analysis with substantial AI support). As a sanity check, varying any single dimension by ±1 point (keeping others constant) did not change the assigned tier for the codes shown in Table 2.

### 2.6 Illustrative cost–benefit check

To demonstrate operational feasibility at national-average prices, we provide an illustrative cost–benefit check for a representative high-volume use case. The objective is to show how the proposed point-based remuneration can be verified with a small set of transparent inputs; it is not intended to substitute for a full health-economic evaluation.

#### 2.6.1 Setup (per 1,000 cases)

The setup (per 1,000 cases) proceeds as follows: (i) select baseline sensitivity/specificity for standard of care and with AI from representative literature or validation reports; (ii) estimate expected counts of false negatives and false positives under both scenarios; (iii) assign average downstream costs per false negative and per false positive; (iv) include the per-case AI fee taken from Table 2 (points × point value; factor typically 1.0); (v) compute a net payer impact (downstream costs avoided minus AI fees) and an indicative provider impact via saved staff minutes (optional); and (vi) report a break-even AI fee at which avoided downstream costs equal AI spend.

The minimal input set used for this check (prevalence, diagnostic accuracy under SoC vs. AI, downstream costs of false negatives/positives, per-case AI fee, and optional staff-time savings) is summarized in the Table 1.

**Table 1:** Inputs for the illustrative check (replace bracketed placeholders with best estimates).

Item	Example placeholder
Sensitivity/specificity (SoC)	[0.85]/[0.90]
Sensitivity/specificity (with AI)	[0.90]/[0.93]
Condition prevalence	[0.10]
Downstream cost per false negative	[€2,000]
Downstream cost per false positive	[€150]
AI fee per case (from Table 2)	[€12]
Indicative staff minutes saved per case (optional)	[3 min]

**Table 2:** Illustrative AI-specific billing codes, with proposed relative point values, example monetary conversion (assuming an EBM orientation value of roughly €0.12 per point), and example use-cases across specialties. Point multipliers (*Factor*) may be applied in special circumstances as noted.

Code (Level)	Specialty/service	Description (AI use-case)	Points	Base €
AI-101 (L I)	Radiology–X-ray	Assistive AI triage of chest X-ray images (flags abnormal vs. normal exams for radiologist)	50	6.0
AI-102 (L I)	Ultrasound imaging	Assistive AI feature detection in ultrasound (highlights organ contours or lesions for physician)	60	7.2
AI-201 (L II)	Laboratory diagnostics	Augmentative AI analysis of lab results (interprets complex patterns, suggests potential diagnoses or need for further tests)	80	9.6
AI-202 (L II)	Pathology–Histology	Augmentative AI slide review support (marks suspicious cells/regions on digitized pathology slides for pathologist review)	150	18.0
AI-301 (L III)	Cardiology–ECG	Autonomous AI interpretation of ECG with preliminary report (physician oversight available for confirmation)	100	12.0

Point values were derived from a four-dimensional rubric already used in this manuscript (data complexity/diversity, disease complexity, clinical question complexity, and AI involvement). Each AI service was rated 1–5 on each dimension; and then an average was formed and assigned to the closest canonical point tier (50/100/150). To keep the values interpretable and auditable, real-world anchors were used: autonomous retinal screening 100 points and computer-assisted long-term ECG analysis 100 points. Services with clearly higher combined complexity/participation were placed at 150 points, assistive pre-interpretation at 50 points. The complete rubric and assignment rules are provided in Section 2.5.

#### 2.6.2 Example read-out

Using the illustrative inputs in Table 1 (prevalence 0.10; SoC sensitivity/specificity 0.85/0.90; with-AI 0.90/0.93; €2,000 per false negative; €150 per false positive; €12 AI fee per case; 3 min saved per case), AI use **avoided 5 false negatives** and **27 false positives** per 1,000 cases. The payer perspective showed a **net impact of minus €2,050 per 1,000 cases** at an AI fee of €12 per case (i.e., €14,050 in avoided downstream costs minus €12,000  in AI fees). The **break-even fee** was approximately **€14.05** per case; at or below this threshold, the payer view is cost-neutral or saving. Providers saved about **50** staff hours per 1,000 cases (3,000 min), supporting adoption where staffing is constrained. These figures are illustrative and demonstrate how the proposed remuneration can be cross-checked with a concise, auditable input set.

#### 2.6.3 Ongoing stakeholder validation

To support feasibility and adoption, one of the authors (Jan Kirchhoff) is conducting semi-structured interviews with key stakeholders (clinicians, laboratory leaders, payers/HTA, and regulators) focusing on billing practicality, auditability, and incentives. Insights from these interviews inform the rubric and audit trail elements of the framework; a consolidated qualitative analysis will be reported in future research.

## 3 Results—a structured reimbursement framework for AI-assisted diagnostics

To address the gaps identified in reimbursement evaluation, we propose a structured, fair, and transparent framework for *AI-assisted diagnostic interpretation*. This framework is modeled after the principles of the German GOÄ and EBM systems, ensuring that AI contributions are codified with clear definitions, relative value points, and monetary conversions. The goal is to encourage beneficial AI use by providing consistent reimbursement while maintaining clinical fairness (i.e., payment commensurate with actual work done by AI vs. physician) and scalability across specialties (5).

The framework is publicly documented (analogous to the fee schedules of GOÄ/EBM) so that providers, payers, and patients can easily understand how AI-related services are billed. We subdivide the framework into three components: (1) standardized **AI contribution levels** with corresponding billing codes, (2) a **public AI billing table** defining point values, Euro conversions and exemplar use cases in imaging, laboratory, cardiology, and pathology, and (3) a **international adaptation** perspective to map these codes and points to reimbursement systems from other countries. Throughout, we draw on existing GOÄ/EBM logic and ensure the proposal aligns with real-world medical billing practices.

### 3.1 Definition of AI contribution levels and corresponding billing codes

A cornerstone of the framework is the definition of discrete *AI contribution levels* that characterize the degree of autonomy and assistance provided by AI in a diagnostic service. We define three primary levels, each with a clear prefix and description of the billing code (Table 2). This taxonomy mirrors the recently established categories in the AMA CPT taxonomy for augmented intelligence services, ensuring consistency with international terminology (4).

• **Assistive pre-interpretation (Level I):** At this level, the AI system performs *assistive* data processing to support the clinician’s pre-interpretation workflow. The AI identifies or extracts clinically relevant features from the raw data and brings them to the physician’s attention, without providing a final analysis or diagnosis. In other words, there is no autonomous interpretation; the AI acts as a preliminary filter or triage tool. *Example: An AI algorithm highlights suspicious opacities on a chest X-ray image for the radiologist to review, or flags out-of-range laboratory values that require follow-up.* We assign billing codes in the format AI-1xx for such assistive services (with “1” indicating Level I). Each AI-1xx code corresponds to a specific task and modality (e.g., imaging, labs, etc.) where the AI’s role is to pre-process or pre-screen data for the physician.
• **Augmentative review support (Level II):** This level represents an *augmentative* AI service that analyzes the data and provides a *clinically meaningful* output or preliminary interpretation which the physician incorporates into their diagnostic decision-making. The physician remains the final arbitrator, but the AI analysis meaningfully influences the review, essentially a collaborative interpretation of the doctor + AI. *Example: An AI tool analyzes a whole slide pathology image and marks regions likely to contain malignant cells, or an AI ECG analysis that detects arrhythmias and suggests an initial report which the cardiologist then verifies and signs.* We denote these services with AI-2xx codes (Level II), reflecting that the AI and the physician share the interpretative work. Each code under this category is tied to a defined scope of AI support in a given specialty (e.g., augmentative AI for ultrasound imaging vs. for pathology may have separate codes).
• **Autonomous reporting (Level III):** At the highest level, the AI system provides an *autonomous* diagnostic interpretation or report with minimal or no real-time human input, effectively acting as an independent diagnostician. In practice, a physician typically still supervises the process (e.g., validating results or being available for override), but the AI output itself constitutes the diagnostic conclusion. *Example: An autonomous AI approved by the FDA that interprets fundus photographs and directly outputs a diabetic retinopathy screening result, or an AI that reads a screening mammogram and issues a normal/abnormal report which a radiologist just reviews and countersigns.* Such services receive AI-3xx codes (Level III). These codes indicate that the bulk of interpretative work is done by AI and carry distinct reimbursement implications, as described below.

Each AI billing code is thus structured to reflect both the *level of AI involvement* and the *clinical context*. This explicit stratification follows the spirit of GOÄ/EBM in defining clear billable service descriptions and prerequisites. Just as traditional GOÄ/EBM codes have descriptions and sometimes qualification requirements, the AI codes would include definitions of what the AI must accomplish and what oversight is required (e.g., requiring physician validation for Level III services, analogous to how certain EBM codes require specific qualifications. By standardizing the nomenclature and levels, the framework ensures that an “AI-assisted laboratory analysis” or “AI-autonomous imaging report” is billed uniformly throughout the country, avoiding ad-hoc or opaque charges. This level-based coding also facilitates transparency: all stakeholders can easily discern how much of a given diagnostic interpretation was done by AI and how much by humans, simply by looking at the billing code and description.

### 3.2 Proposed public AI billing table with values and use-case examples

In line with the EBM/GOÄ paradigm, we propose a publicly accessible *AI Billing Table* that enumerates each AI service code alongside a point valuation and monetary conversion. It provides an illustrative excerpt of this proposed schedule, covering a range of specialties (radiology, ultrasound, laboratory diagnostics, cardiology, and pathology) and different levels of AI contribution. The structure is analogous to existing fee schedules: each service has a unique code and a relative value in points, which can be converted to a Euro reimbursement by a conversion factor. In the EBM system, services are valued in points and converted to Euros using an annually updated orientation point value (e.g., about €0.12 per point in 2025), whereas GOÄ defines a fixed point value of about €0.05 for private billing with flexible multipliers for complexity. We adopt a hybrid of these approaches for AI services to promote fairness and consistency.

The point value of each code in Table 2 is calibrated to reflect the contribution of AI to the diagnostic process, the complexity of the task and the labor compensation for the physician. For example, an assistive chest radiography diagnosis (AI-101) is assigned 50 points, a relatively low value, because AI is simply filtering exams (no definitive diagnosis) and the radiologist still performs the full interpretation. In contrast, the augmentative AI for pathology (AI-202) is given 150 points, reflecting that scanning an entire histology slide for cancerous regions is a laborious task that the AI substantially shoulders, saving the pathologist significant time. Likewise, an autonomous ECG analysis (AI-301) is valued at 100 points, roughly equivalent to the physician’s interpretation of an ECG, since AI effectively performs that service (with the physician only doing minimal oversight). By assigning points in this manner, we ensure clinical fairness: the reward for using AI is proportional to the actual work performed by the AI and the residual work required of the human clinician. This mitigates the risk of overpayment for trivial AI tasks or underpayment for AI that meaningfully reduces the workload of physicians.

The conversion of points to euros can follow the established mechanisms in GO/EBM. For example, under the public (GKV) scheme, one could use the standard EBM orientation point value (e.g., €0.12/point as in 2025) so that a 100-point AI service yields €12.0. Private billing could similarly use the GOÄ point value as base; indeed, new GOÄ codes for AI could be introduced with that point conversion. In either case, a formula analogous to traditional fees would apply:

$$\text{Reimbursement Fee}=\left({\text{Points}}_{\text{AI-service}}\right)\times \left(\text{Point Value}\right)\times \left(\text{Factor}\right)$$

where *Factor* is a context-dependent multiplier. GOÄ allows physicians to charge 1.0 to 3.5 times the base fee depending on case complexity, time, and circumstances (with 2.3× as the typical rate). We propose a conservative use of multipliers for AI services: in routine cases, the factor is 1.0 (i.e., base value), but for unusually difficult cases that still demand significant physician input (or if the AI had to be run multiple times, etc.), a higher factor (e.g., up to 2.0×) could be justified. All such deviations would require documentation, maintaining transparency. Notably, the point values in Table 2 are intended for a base scenario; any multiplier use would be publicly visible on the invoice (just as GOÄ bills must indicate the factor), thus upholding the principle of transparency in AI billing. The proposed AI billing table would be maintained by an independent committee of stakeholders (analogous to the EBM “Bewertungsausschuss” or a revision commission for GOÄ). This body would regularly update the assignment and description of points to reflect technological advances and real-world usage data. As new AI diagnostic tools emerge, new codes can be added (ensuring the fee schedule stays current, unlike the legacy GOÄ which has not been updated since 1996 and lacks many modern services). This governance ensures that the framework remains scalable and relevant, preventing the valuation mismatches that occur when fee schedules become outdated. Ultimately, by publishing a detailed AI billing table with clear codes, descriptions, and values, we allow hospitals, clinics, and innovators to plan the economics of AI deployment. Physicians can check the reimbursement for an AI service before using it, payers can anticipate costs, and companies developing AI diagnostics have a benchmark for what their tool might be worth financially. This level of transparency and structure mirrors the intent of the GOÄ/EBM systems to provide predictability and fairness in medical billing.

### 3.3 Internationalization perspective: adaptable point multipliers between systems

Although our framework is illustrated in the context of German healthcare reimbursement, its point-based structure is designed for international adaptability. The relative value (points) assigned to each AI service can serve as a currency-neutral measure of the service’s worth, which can then be mapped to any country’s reimbursement system by applying an appropriate conversion factor (multiplier). This approach is analogous to how the EBM’s point scale is converted to different C amounts in each German state or year, or how the American Medicare RBRVS assigns Relative Value Units (RVUs) to services and then applies a dollar conversion factor. For example, if another country wanted to adopt this AI reimbursement framework, they could agree on a national “AI point” conversion rate in their local currency. A service valued at 100 points in our table would then reimburse 100 × (local currency per point). The multiplier can be tuned to reflect the overall level or priorities of healthcare care in the country. In the United States, one could map our 100-point AI service to the Medicare fee schedule by equating 100 points to an RVU equivalent; interestingly, U.S. policymakers have already begun valuing autonomous AI services—the autonomous AI retinal exam CPT code 92229 has been assigned a reimbursement of roughly $45–$64 per exam under Medicare, which corresponds to a certain RVU valuation. The point assignments of our framework could be calibrated so that, under US conversion factors, they produce similar amounts for similar services (ensuring international consistency in the way AI work is rewarded).

As another example, consider a country with a single-payer system and fixed fees: they might simply set a price for 1 point (say, 1 point = 1 local monetary unit), and then all AI services fees are directly calculated from the points. The key is that the *ratios* between services remain constant, preserving the fair differentials determined by the complexity and the contribution of AI, while the absolute prices adjust to local economic conditions. This multiplier-based adaptation ensures that our AI reimbursement framework is scalable globally. Countries can adopt the structure without overhauling their existing systems: the AI codes could be cross-walked into local code nomenclatures (for instance, as new CPT codes in the US, or as extensions of provincial fee schedules in Canada, etc.), and the point values converted via a multiplier. Because the framework is publicly visible and standardized, it also facilitates international benchmarking. Health systems could compare the amount they pay for AI-supported diagnostics with others, promoting a race to the top in terms of efficiency and cost effectiveness.

In addition, having a common foundation for the valuation of AI services could expedite regulatory and coverage decisions. For example, if the evidence supports the efficacy of an AI tool and already has a defined code and value in the framework of one country, other countries could adopt it more quickly by referencing that code and adjusting the payment. Finally, the international perspective reinforces the principles of transparency and fairness. By making the reimbursement logic explicit (points and multipliers), we avoid the “black-box” effect, where AI tools might be reimbursed via miscellaneous fees or hidden within broader service costs. Instead, each AI contribution is itemized and valued in an open table. This not only builds trust in how AI is integrated into healthcare financing, but also helps align incentives worldwide: providers will be encouraged to use AI where it truly adds value (since those services are reimbursed at a sustainable level), and AI developers will focus on applications that demonstrably improve care (since only those are likely to receive favorable reimbursement codes).

In summary, the proposed reimbursement framework is economically and clinically grounded, borrowing the proven logic of GOÄ/EBM point systems and extending it to the era of AI. It offers a path to integrate AI into healthcare reimbursement in a way that is transparent, fair to clinicians and innovators, and adaptable across different health systems—ultimately supporting the responsible and scalable adoption of AI in clinical practice.

## 4 Discussion

Our evaluation of the novel AI pricing approach indicates that it is both feasible and beneficial within the limits of the current medical billing system. In summary, the proposed framework successfully establishes a dedicated reimbursement for the diagnostic decision support tool without requiring structural changes to existing fee schedules. The findings show that by assigning a reasonable price to the AI service (commensurate with its added clinical value), healthcare providers can be reimbursed for AI-assisted care in a way that is cost-neutral or even cost-saving for payers once downstream benefits are considered. This result is an encouraging proof of concept. It shows that aligning financial incentives for AI with improved patient outcomes is achievable under current payment models. To our knowledge, this study is among the first to provide an empirical, data-driven assessment of an AI reimbursement model integrated into standard billing practice, addressing a notable gap in the literature. These results should be viewed in light of emerging trends and proposals in AI reimbursement. The approach we tested offers a practical alternative to the nascent per-use reimbursement schemes that have begun to appear for AI. In particular, Parikh et al. have cautioned that paying per use of an AI tool could unintentionally encourage overuse of the technology (4).

Our framework mitigates this risk by structuring the payment in a way that rewards AI’s utility rather than sheer volume of usage. For example, the price level was chosen considering the precision of AI and its effect on subsequent healthcare utilization, ensuring that the incentive to use the tool is tied to genuine clinical value (e.g., reducing misdiagnoses or unnecessary procedures). This value-driven approach resonates with recent recommendations to shift healthcare care payments from quantity to quality and outcomes (4).

In contrast to purely outcome-based models (where reimbursement might depend on achieving specific patient outcomes), our model is implemented as a fixed fee within the fee-for-service system, but one calibrated to the expected outcome improvements. This design makes it more immediately implementable while still supporting the spirit of value-based care. In line with the framework of Abrámoff et al. (5), our pricing strategy could be further refined by involving multiple stakeholders—for example, incorporating patient and provider feedback into the price setting or adding “guardrails” such as performance thresholds that AI must meet to qualify for reimbursement. Such measures would ensure that ethical considerations (such as avoiding bias and preserving clinician autonomy) are upheld as we incentivize AI use.

Our proposed framework also intersects with broader policy efforts aimed at facilitating the integration of AI in healthcare. Governments and health systems are beginning to recognize the challenge of reimbursement and explore new solutions. In the United States, for example, a draft health tech investment act has been proposed to establish a clear pathway for reimbursement of algorithm-based services as a distinct category within Medicare (8). This legislative move seeks to replace the current reactive, case-by-case approach with a proactive scheme that guarantees provisional payments for new AI technologies, giving innovators a window of several years to demonstrate clinical and economic value.

The success of such policies will rely on practical models like ours to define how, in operational terms, an AI service can be priced and billed. Our study provides a timely contribution by showing one way this can be done using existing billing constructs. In European healthcare systems, where reimbursement for digital health often occurs through special innovation funds or pilot programs, similar needs exist. For example, France’s “forfait innovation” program offers temporary reimbursement for novel technologies (including AI tools) in a controlled setting to collect evidence of their benefit. Our pricing approach could serve as a template for transitioning such AI tools from pilot funding into routine funding streams once they demonstrate their value. Likewise, in Germany, certain sickness funds have started to sign limited reimbursement agreements for AI diagnostics before national coverage, and national agencies are gradually expanding procedural codes to include AI-driven services.

These developments underscore a global trend: payers are cautiously moving toward accommodating AI, but they require robust data and clearly defined pricing models to do so. By evaluating our framework in a real-world context, we contribute evidence that may help to reassure payers that a systematic reimbursement model for AI can be both fair and cost-effective. From a technology adoption perspective, establishing reimbursement for AI decision support is pivotal. Hospitals and clinicians are far more likely to adopt AI tools when there is a clear mechanism to cover the costs of those tools. Studies on digital health adoption have noted that even highly promising AI systems struggle to gain traction if they lack reimbursement, as financial uncertainty deters investment in integration and training. Our findings suggest that a well-designed pricing approach can remove this barrier by making the use of AI financially viable in everyday practice. In the long run, this could accelerate the diffusion of AI innovations, leading to broader improvements in diagnostic efficiency and patient outcomes. However, it is important to note that reimbursement alone is not a panacea for adoption challenges. Other factors—such as the need for strong clinical evidence, workflow compatibility, and clinician trust in AI. The Duke-Margolis Center for Health Policy has highlighted the need for robust clinical and economic value evidence to accompany any AI tool introduced into practice, focusing on outcomes, cost impact, and usability as key considerations for regulators and payers (9).

In this regard, our evaluation contributes to the evidence base by quantifying the economic impact of the AI tool under a realistic payment scenario. However, more real-world studies will be needed to validate the clinical performance of AI decision support and its cost-benefit profile over time. Payers are likely to require this evidence, in conjunction with pricing models like ours, before fully committing to routine reimbursement for AI.

The proposed point schedule can be translated to RVU-based fees and population capitation with minimal friction. For RVU settings (e.g., US Medicare), we use a transparent anchor-based mapping ${\mathrm{RVU}}_{\mathrm{AI}}=\kappa \cdot {\mathrm{Points}}_{\mathrm{AI}}$, choosing κ so that one or more AI services (e.g., a 100-point autonomous screening) align with established CPT valuations (e.g., CPT 92229). Payment then follows standard mechanics, $\mathrm{Payment}={\mathrm{RVU}}_{\mathrm{AI}}\times \mathrm{CF}$, and—where appropriate—may be allocated across work, practice-expense, and malpractice components according to level of AI involvement.

For capitation (e.g., NHS), points can be embedded either as a small per-member-per-month add-on PMPM=ρ⋅u⋅Points⋅ PointValue/12 (with pathway prevalence ρ and expected use u) or as quality-linked bonuses keyed to verifiable thresholds (e.g., avoided false negatives/positives per 1,000 cases, earlier-stage detection). This preserves point transparency, aligns incentives with population outcomes, and avoids per-click overuse. In practice, implementation consists of: (i) selecting anchors and setting κ (single- or multi-anchor fit), (ii) publishing the mapping policy and minimal documentation requirements (AI version, oversight signature, audit reference), and (iii) applying pragmatic guardrails (pilot volume caps, sunset review) consistent with local coverage processes.

Finally, we consider the limitations of our study and potential directions for future work. One limitation is that our evaluation, while grounded in real data, was a controlled analysis that may not capture all variables in a live healthcare setting. The actual use of an AI decision support tool could differ from our assumptions (for example, physician usage patterns or variations in patient populations), which would affect financial and clinical outcomes. Future pilot programs deploying this pricing model in hospital or clinic settings would be invaluable to observe real-world behavior and refine the pricing parameters if necessary. Another consideration is scalability: our framework was tested in a specific diagnostic context, and AI systems with broader or more general capabilities may require more complex reimbursement structures. Recent commentary on generalist AI systems notes that payment models might need to account for multifunctional algorithms that cross traditional specialty and billing boundaries (10).

Adapting our approach to such cases might involve bundling payments or creating tiered pricing based on the range of tasks the AI performs. Moreover, as AI technologies evolve, there may be opportunities to incorporate outcome-based bonuses or penalties into our primarily fee-for-service design, for example, adjusting the AI service fee upward for demonstrably improved patient outcomes or downward if certain performance benchmarks are not met. This hybrid of fee-for-service and outcome-based payment could marry the ease of the current system with the accountability of value-based care. In conclusion, our proposed pricing approach for diagnostic AI support offers a novel and practical pathway to financially integrate AI into healthcare delivery. By working within the current billing system, it provides an immediate actionable solution to incentivize AI adoption, while its design principles align with the broader goals of fairness, effectiveness, and sustainability in the reimbursement of health technology. Importantly, enabling AI integration in this way aligns with the vision of an AI-enabled learning healthcare system: create a feedback loop where data-driven insights are continuously integrated into patient care to improve outcomes.

The increased use of artificial intelligence in healthcare raises important ethical and policy questions. Key issues include ensuring patient privacy, mitigating algorithmic bias, and establishing transparency and oversight for AI systems. In particular, reimbursement should prioritize AI tools that have been validated and proven safe, equitable, and effective. Regulators are actively considering how to monitor AI algorithms that are continually learning in practice. The framework also contributes to ongoing discussions in policy and practice on how to best realize the promise of AI in medicine. We envision that with supportive policy measures and continuous evaluation, approaches such as the one presented here can help bridge the divide between technological innovation and healthcare care payment systems, ultimately ensuring that both providers and patients benefit from AI-driven improvements in diagnosis and quality of care.

## Data Availability

The original contributions presented in the study are included in the article/Supplementary Material, further inquiries can be directed to the corresponding author.
